# The Effect of *Cucumaria frondosa* Tentacles Hydrolysates on Dextran Sulfate Sodium-Induced Colitis: Integrated Metagenomics and Metabolomics Analysis

**DOI:** 10.3390/foods14203483

**Published:** 2025-10-13

**Authors:** Senyu Zhang, Qiuting Wang, Shunmin Gong, Mingbo Li, Yu Zhang, Leilei Sun, Liqin Sun

**Affiliations:** Yantai Key Laboratory of Characteristic Agricultural Bioresource Conservation & Germplasm Innovative Utilization, School of Life Sciences, Yantai University, Yantai 264005, China; zsy10698811@163.com (S.Z.); wqt1981979471@163.com (Q.W.); g18807041394@163.com (S.G.); limingbo1711@163.com (M.L.); sliqin2005@163.com (L.S.)

**Keywords:** *Cucumaria frondosa* tentacles hydrolysates, inflammatory bowel disease, gut microbiota, multi-omics, nutraceutical

## Abstract

Inflammatory bowel disease continues to pose substantial therapeutic challenges in modern gastroenterology. This study systematically evaluated the anti-colitis efficacy of *Cucumaria frondosa* tentacles hydrolysates (CFTHs) using a dextran sulfate sodium (DSS)-induced murine colitis model. Characterized by enhanced stability and solubility with molecular weights below 1000 Da, administration of CFTHs demonstrated a significant mitigation in colitis pathology. Therapeutic outcomes included an improved splenic index, attenuated colonic mucosal damage, and substantial decreases in serum pro-inflammatory cytokines. Relative to the DSS group, the MPO value in the CFTHs-H group decreased by 27.6%, and the IL-6 value exhibited a reduction of 33%. Metagenomic profiling revealed that CFTHs mediated gut microbiota modulation, particularly the enrichment of beneficial *Bacteroidetes* and suppression of pro-inflammatory *Proteobacteria*. Metabolomic analysis identified elevated colonic concentrations of anti-inflammatory metabolites such as gamma-linolenic acid and prostaglandin I2, suggesting a microbiome–metabolome crosstalk in the therapeutic mechanism. These multi-omics findings in a murine model suggest that CFTHs may represent a promising candidate for future studies as a nutraceutical intervention for inflammatory bowel disorder. This intervention may operate through mechanisms that include simultaneous immunomodulation, microbiota restoration, and metabolic reprogramming.

## 1. Introduction

Sea cucumbers are marine invertebrates recognized for their significant nutritional richness and potential health benefits, which are particularly attributed to their elevated protein content. *Cucumaria frondosa* is a prevalent species found in northern waters, primarily residing in frigid environments of the North Atlantic and the Barents Sea along the Russian coast [1]. This slow-growing species inhabits temperate to polar regions, spawning annually and accumulating nutrients within its body [2].The processing of sea cucumbers generates a significant amount of by-products, such as tentacles and internal organs (gonads, respiratory tracts, and intestines), which are often discarded, leading to substantial resource waste. It has been demonstrated that *Cucumaria frondosa*’s body wall serves as a natural source of various bioactive compounds, including anticancer agents and anti-angiogenic, anti-inflammatory, anti-diabetic, antioxidant, and antimicrobial substances [3]. Notably, in our previous studies, the tentacles of *Cucumaria frondosa* were found to be abundant in protein, with a concentration of up to 57.1% [4]. Although some studies have been conducted on the body wall or viscera of *Cucumaria fructosa*, research focusing on its tentacles, particularly its protein hydrolysates (CFTHs), remains relatively limited.

Chronic inflammatory disorders, such as ulcerative colitis (UC) and Crohn’s disease (CD), are collectively termed inflammatory bowel disease (IBD) [5]. The etiology of UC is multifaceted, involving viral, microbial, and environmental factors that perpetuate immunomodulatory dysfunction in genetically susceptible individuals [6]. Currently, aminosalicylates, immunosuppressants, and corticosteroids are employed to mitigate the inflammation and immune response associated with UC, aiming to provide symptomatic relief [7]. However, the long-term administration of current therapeutic drugs is associated with considerable adverse effects. This underscores the necessity of identifying new active compounds that offer enhanced safety for the treatment of UC [8]. A substantial body of research has highlighted the relationship between gut microbiota dysbiosis and UC, generating increasing interest in enhancing gut microbiota composition as a therapeutic strategy for colitis [7,9].

Owing to their unique biological activities, marine-derived protein hydrolysates have emerged as compelling candidates for clinical applications. The preparation of these hydrolysates, whether through chemical or enzymatic means, results in a heterogeneous composition of peptides and free amino acids. Utilizing enzymatic processes is an efficient approach for generating protein hydrolysates and recovering bioactive peptides which exhibit health-promoting and disease risk-reducing capacities [10]. Multiple studies have demonstrated that protein hydrolysates possess a wide range of biological functions without adverse effects, including antithrombotic, anticancer, antihypertensive, anti-inflammatory, immunomodulatory, antimicrobial, and antioxidant activities [11,12]. A growing body of research points to the amelioration of DSS-induced colitis by quinoa protein and its hydrolysates is mediated via the inhibition of the TLR4/IκB-α/NF-κB pathway and the modulation of gut microbiota composition [13]. Additionally, sea cucumber peptides exert anti-inflammatory effects in the murine colon via modulation of the miR-155/SOCS1 axis [14]. Strong anti-inflammatory activity has also been demonstrated for Frondano, an intestinal extract from the Atlantic sea cucumber [15]. However, the alterations in the intestinal microbiota of mice with colitis, resulting from hydrolysates derived from the tentacles of *Cucumaria frondosa*, and the associated metabolome-related mechanisms remain undocumented.

This research utilized tentacles from the by-products of *Cucumaria frondosa* to produce CFTHs through protease hydrolysis. The molecular weight distribution and surface hydrophobicity were determined to identify suitable proteases and to elucidate the characteristics of CFTHs. Concurrently, DSS treatment was employed to induce colitis, serving as a standard model for UC. The improvement of DSS-induced colitis by CFTHs might be related to changes in gut microbiota structure, consistent with the restoration of immune homeostasis. To test this hypothesis, this study was designed with the following specific objectives: to evaluate the protective efficacy of CFTHs against DSS-induced colitis by assessing clinical symptoms, gut barrier integrity, and inflammatory responses; to characterize the impact of CFTHs on the gut microbial composition using metagenomic sequencing; to profile the shifts in the host metabolome induced by CFTHs treatment using untargeted metabolomics; and to integrate multi-omics datasets to elucidate potential correlations between microbiota remodeling and metabolic changes. *Cucumaria frondosa* tentacles, as a by-product of the existing sea cucumber processing industry, provide a sustainable and cost-effective raw material. Their high protein content contributes valuable components for hydrolysate preparation. The enzymatic hydrolysis process employed is a well-established, scalable, and environmentally friendly technology in the fields of food and biotechnology, enhancing its potential for industrial applications.

## 2. Materials and Methods

### 2.1. Experimental Materials and Reagents

*Cucumaria frondosa* tentacles were obtained from Haizhongbao Seafood Trading Center (Yantai, China). Flavourzyme (15,000 U/g) was obtained from Beijing Solarbio Biotechnology Co., Ltd. (Beijing, China). Mesalazine (≥98.5% purity) was acquired from Heilongjiang Tianhong Pharmaceutical Co., Ltd. (Meishaxin, Harbin, China). DSS (MW: 36,000−50,000 Da, Cas: 9011-18-1, LOT: j07310) was sourced from Dalian Meilun Biotechnology Co., Ltd. (Dalian, China), while the fecal occult blood test kit (R21607-300T, LOT: J27IR221326) was supplied by Shanghai Yuanye Biotechnology Co., Ltd. (Shanghai, China). Additionally, enzyme-linked immunosorbent assay (ELISA) kits for mouse tumor necrosis factor-alpha (TNF-α), interleukin-6 (IL-6), interleukin-1 beta (IL-1β), and myeloperoxidase (MPO) were obtained from Quanzhou Jiubang Biotechnology Co., Ltd. (Quanzhou, China). The OMEGA Mag-Bind Soil DNA Kit was acquired from Omega Bio-Tek, Inc. (Norcross, GA, USA), while the Illumina TruSeq Nano DNA LT Library Prep Kit was obtained from Illumina, Inc. (San Diego, CA, USA). All remaining chemicals adhered to analytical-grade standards.

### 2.2. Preparation of CFTHs

The tentacles of *Cucumaria frondosa* were washed with distilled water. Subsequently, the tentacles were lyophilized for 48 h and then crushed. Four distinct proteases were employed to conduct the hydrolysis under their respective optimal conditions (Table 1) [4]. Following inactivation through boiling in water for 15 min, the mixture was allowed to cool to room temperature. To obtain the supernatant, the solution was subjected to centrifugation at 11,000× *g* for 15 min at 4 °C. The final product of CFTHs was obtained through a subsequent lyophilization process.

### 2.3. Structural and Physicochemical Characterization

#### 2.3.1. Surface Hydrophobicity

According to the method established by Zhang et al. [16], the surface hydrophobicity (H_0_) was determined using the hydrophobic probe 1-anilino-8-naphthalenesulfonate (ANS). The H_0_ was assessed after diluting each sample to a concentration of 1 mg/mL in 0.1 M phosphate-buffered saline (PBS) at pH 7.0. Subsequently, 20 μL of an 8 mM ANS solution was added to 2.0 mL of the diluted sample and mixed thoroughly. The fluorescence value at the emission wavelength was measured via fluorescence spectrophotometer (Shimadzu, Tohoku, Japan). A regression curve was generated to establish the relationship between fluorescence intensity and protein concentration, with the slope indicating the surface hydrophobicity of the CFTHs.

#### 2.3.2. Molecular Weight Distribution

High-performance liquid chromatography (Waters, Milford, MA, USA) was employed to determine the molecular weight distribution of the hydrolysates, as described by Yu et al. [17]. A relative molecular mass calibration curve was established using standard substances, including Gly-Gly-Gly (MW 189 Da), Gly-Gly-Tyr-Arg (MW 451 Da), VB12 (MW 1355 Da), and aprotinin (MW 6511.44 Da).

### 2.4. Animals and Experimental Design

Male C57BL/6J mice (7 weeks old) were procured from Jinan Pengyue Laboratory Animal Breeding Co., Ltd. (Jinan, China). In compliance with the Guide for the Care and Use of Laboratory Animals of Yantai University, all animal experiments in this study were conducted and received approval from the Ethics Committee. Throughout the study, all mice were housed under controlled conditions (25 °C, 40−60% humidity). Ambient lighting was supplied from 7 AM to 7 PM (12 h of light and 12 h of darkness), with food and water available ad libitum.

**Figure 1:**
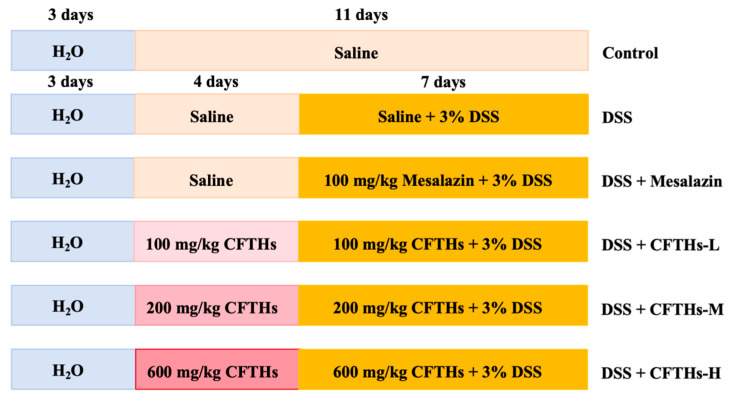
Schematic diagram of the DSS-induced colitis modeling and treatment cycle.

Sixty healthy mice (18.5 ± 1.0 g) were randomly assigned to six groups (ten mice per group): Control group (Control), DSS group (DSS), mesalazine-treated group (Mesalazine), CFTHs low-dose group (CFTHs-L), CFTHs medium-dose group (CFTHs-M), and CFTHs high-dose group (CFTHs-H). Following the induction of colitis by administering 3% DSS for 7 days to all groups except the control, therapeutic interventions were initiated concurrently. Mesalazine was administered daily to the positive control group at a dose of 100 mg/kg, while mice in the colitis model were administered CFTHs at low (100 mg/kg), medium (200 mg/kg), or high (600 mg/kg) doses (Figure 1). Doses of CFTHs (100, 200, and 600 mg/kg) were selected based on effective ranges reported in the literature for analogous interventions in murine models [18,19,20]. This selection encompasses a wide range to assess the potential dose–response effect.

Daily body weight measurements were conducted for all mice. At the trial endpoint, the animals were humanely euthanized via cervical dislocation. Subsequently, the serum and organs (livers, spleens, and kidneys) were collected in a sterile environment and weighed promptly. The spleen index (mg/g) was defined as the ratio of spleen weight (mg) to body weight (g). The length of the colon was recorded, and the contents of the colon were immediately collected and stored in an ultra-cold freezer at −80 °C. Additionally, representative colon sections were collected and preserved in 4% paraformaldehyde for further studies.

### 2.5. Disease Activity Index (DAI)

During the feeding period, daily data on the body weights of mice were collected, and stool consistency was evaluated. The presence of diarrhea, stool bleeding, and body weight loss reflected the severity of colitis, as depicted in Table 2. Daily body weight loss was determined as a percentage of the initial weight (day 0). The positivity for bleeding was determined using a fecal occult blood assay test kit. The DAI was derived from a combined assessment of body weight loss, diarrhea, and stool bleeding [21].

DAI = (body weight loss score + stool bleeding score + diarrhea score)/3

### 2.6. ELISA

After homogenization, the supernatant from the colonic tissue was collected via centrifugation for ELISA detection. To quantify the levels of TNF-α, MPO, IL-1β, and IL-6, mouse colon homogenates were analyzed using ELISA kits according to the manufacturer’s protocols.

### 2.7. Hematoxylin–Eosin (H&E) Staining

Following fixation in a 4% paraformaldehyde solution, colonic tissues were routinely processed, which included dehydration, paraffin embedding, and sectioning at 5 μm. Following deparaffinization and rehydration, tissue sections were stained with H&E for histological assessment under a light microscope (OLYMPUS, Tokyo, Japan) [22].

### 2.8. Microbiome Profiling via High-Throughput Sequencing

The isolation of microbial genomic DNA from all samples was conducted following the manufacturer’s protocol for the OMEGA Mag-Bind Soil DNA Kit. Following extraction, the purified DNA samples were cryopreserved at −20 °C to ensure stability until subsequent analytical procedures. DNA concentration was measured using a Qubit™ 4 Fluorometer (WiFi: Q33238, Qubit™ Assay Tubes: Q32856, Qubit™ 1X dsDNA HS Assay Kit: Q33231) (Invitrogen, Carlsbad, CA, USA), while integrity was evaluated through agarose gel electrophoresis with SYBR Safe staining. Metagenomic shotgun libraries were prepared from the microbial DNA using the Illumina TruSeq Nano DNA LT Library Prep Kit, incorporating 400 bp insert fragments. Libraries underwent paired-end sequencing (PE150) on an Illumina NovaSeq platform at Personal Biotechnology Co., Ltd. (Shanghai, China). Analysis commenced with the processing of raw sequencing data using the GenesCloud bioinformatics platform (https://www.genescloud.cn (accessed on 25 April 2025)).

### 2.9. Non-Targeted Metabolomics

In this study, mouse colonic tissue samples (25 mg) were weighed into EP tubes on ice. Following this, 500 μL of extraction solution and homogenizing beads were added. The samples underwent vortexing (30 s), followed by homogenization (35 Hz, 4 min), and subjected to three cycles of sonication (5 min per cycle) at 4 °C. Protein precipitation was achieved by storing the samples at −40 °C with a duration of 1 h. Subsequently, the samples underwent centrifuging at 13,000× *g* for 15 min at 4 °C, and the supernatant was collected in a scintillation vial for analysis. For quality control, a pooled QC sample was generated from equal aliquots of all sample supernatants. Metabolite separation was performed using a Vanquish (Thermo Fisher Scientific, Waltham, MA, USA) ultra-performance liquid chromatograph (UPLC) system fitted with a Waters ACQUITY UPLC BEH Amide column (2.1 mm × 50 mm, 1.7 μm). The mobile phases consisted of 25 mmol/L ammonium acetate/ammonia in water (A) and acetonitrile (B), with the autosampler maintained at 4 °C and a 2 μL injection volume. Mass spectrometry data were acquired on an Orbitrap Exploris 120 instrument running control software (Xcalibur, Version 4.4, Thermo). The subsequent data analysis pipeline included pre-processing, experimental QC, metabolite identification, and comparative analysis between groups.

### 2.10. Statistical Analysis

The animal study employed a completely randomized design. Mice were randomly assigned to one of six groups (n = 10 each): (1) Control group, (2) DSS group, (3) Mesalazine group, (4) CFTHs low-dose (100 mg/kg) group, (5) CFTHs medium-dose (200 mg/kg) group, and (6) CFTHs high-dose (600 mg/kg) group. All data were expressed as the mean ± standard deviation (SD), with n ≥ 3. Statistical analyses were performed using GraphPad Prism software (version 10.6.1, GPS-7772778-GYZA-20BFD, https://www.graphpad.com (accessed on 12 March 2025)) under an institutional site license. Student′s *t*-tests were utilized to evaluate the statistical significance of basic physical biomarkers between the two groups. Differences in experimental data were assessed using one-way ANOVA in IBM SPSS Statistics (version 27, International Business Machines Corporation, https://www.ibm.com/products/spss-statistics (accessed on 16 March 2025)), followed by Tukey’s post hoc test for multiple comparisons. Statistical significance is denoted by * *p* < 0.05, ** *p* < 0.01, *** *p* < 0.001, **** *p* < 0.0001, and ns = not significant.

## 3. Results and Discussion

### 3.1. Structural Characteristics and Physicochemical Properties of CFTHs

#### 3.1.1. Surface Hydrophobicity of CFTHs

**Figure 2:**
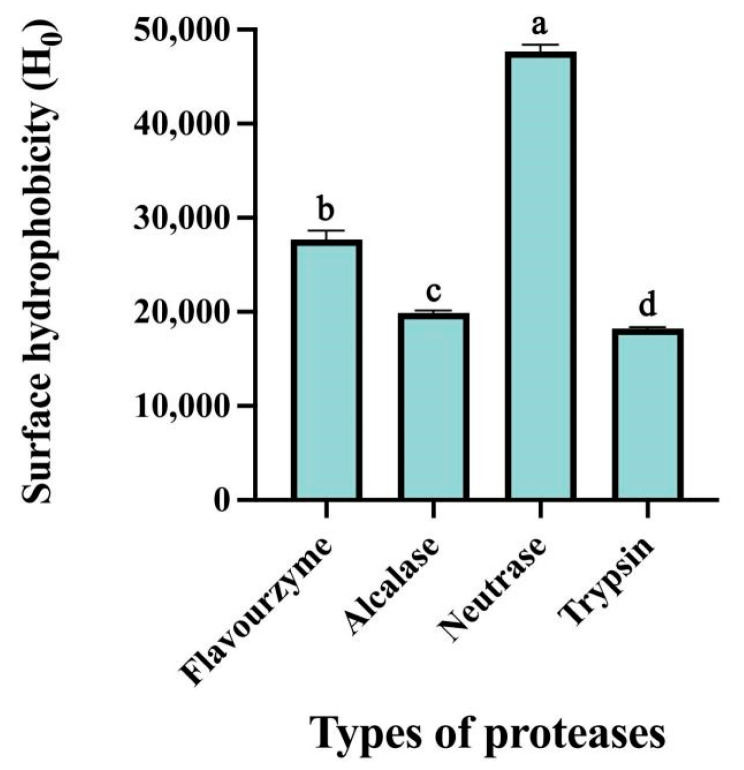
Surface hydrophobicity of hydrolysates prepared using various proteases. Vertical bars represent the standard deviation (n = 3). Different letters indicate significant differences (*p* < 0.05).

ANS can be utilized as an exogenous fluorescent marker to reflect the surface hydrophobicity of hydrolysates. The hydrophobicity of proteins plays a crucial role in their biological activity and technical characteristics, making it particularly significant in the food industry [23]. As illustrated in Figure 2, variations in the surface hydrophobicity of the hydrolysates were observed across the different proteases employed. Notably, neutrase produced the most hydrophobic hydrolysate, a result attributed to its specific enzymatic action and the extent of hydrolysis. These factors dictate the final amino acid composition, which is a key determinant of the differences in the functional properties of CFTHs [24]. Neutrase primarily targets the amino termini of hydrophobic amino acids, like leucine, phenylalanine, and tyrosine. This specificity leads to the exposure of hydrophobic amino acid residues on the molecular surface following enzymatic digestion, thereby significantly enhancing surface hydrophobicity [25]. Furthermore, compared to enzymes that achieve higher degrees of hydrolysis, such as flavourzyme, neutrase exhibits a lower degree of hydrolysis, resulting in the production of peptide fragments with larger molecular weights. This relatively limited hydrolysis prevents excessive cleavage that could degrade hydrophobic groups into smaller peptides or amino acids, thereby preserving a greater number of hydrophobic moieties [26].

#### 3.1.2. Molecular Weight Distribution of CFTHs

**Figure 3:**
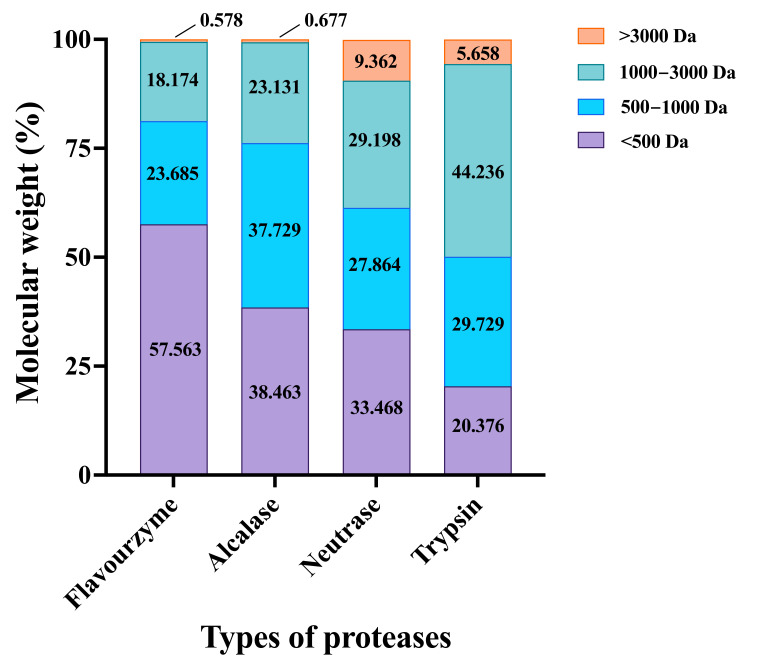
Molecular weight distribution of hydrolysates prepared using various proteases.

The molecular weight distribution of CFTHs hydrolyzed by four different proteases was illustrated in Figure 3. The molecular weight of peptides is closely related to their antioxidant activity, with lower-molecular-weight components typically exhibiting stronger antioxidant properties [27]. The hydrolysates produced by favourzyme, alcalase, neutrase, and trypsin comprised mixtures of peptides with varying molecular weights. Specifically, the proportions of polypeptides with a relative molecular mass below 500 Da were 57.563%, 38.463%, 33.468%, and 20.376% for favourzyme, alcalase, neutrase, and trypsin, respectively. Overall, favourzyme hydrolysates demonstrated a higher concentration of small peptides compared to the other three hydrolysates, suggesting that favourzyme was more effective in generating small peptides [17]. Therefore, favourzyme was selected for the enzymatic hydrolysis of *Cucumaria frondosa* tentacles to prepare hydrolysates for subsequent evaluation of anti-inflammatory activity.

### 3.2. CFTHs Alleviated the Symptoms of DSS-Induced Colitis

**Figure 4:**
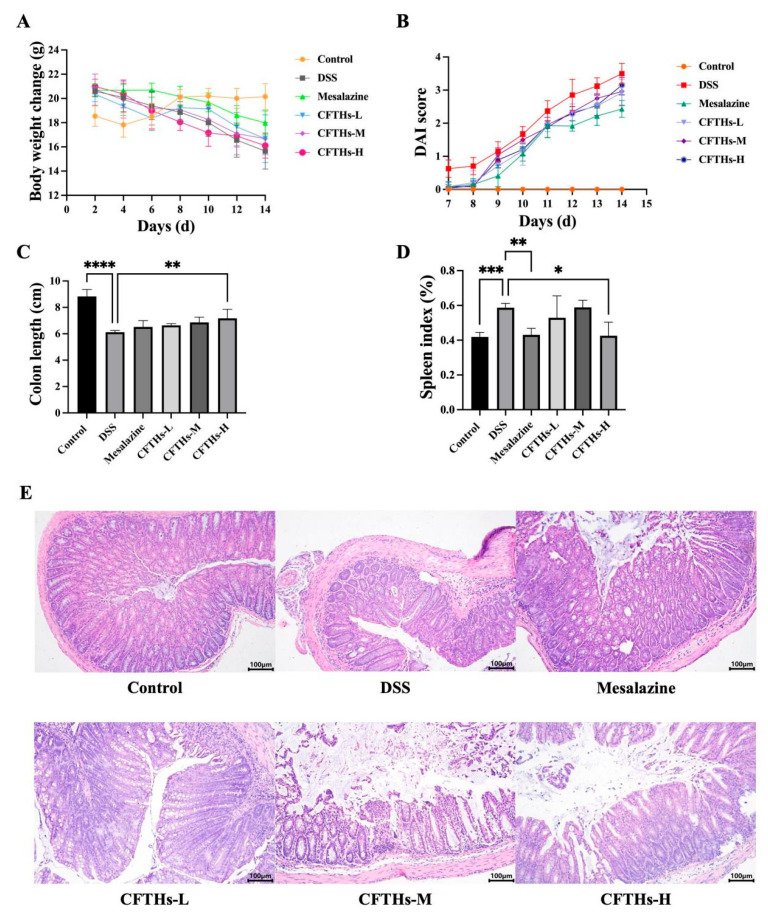
Assessment of colitis severity induced by DSS. (**A**) Body weight; (**B**) DAI score; (**C**) colon length; (**D**) spleen index; (**E**) representative H&E-stained colon sections (100 × magnification). Vertical bars represent the standard deviation (n = 10); statistical analyses were conducted using one-way ANOVA followed by Tukey’s post hoc test. “*” denotes statistically significant differences with * *p* < 0.05, ** *p* < 0.01, *** *p* < 0.001, and **** *p* < 0.0001.

Weight loss, shortened colon length, and elevated DAI values are the primary symptoms associated with colitis. Disturbances in colon function can lead to typical manifestations, including periods of diarrhea and constipation, often accompanied by bloating. Therefore, weight loss is frequently utilized as an indicator of the clinical status of colitis [28]. All groups treated with mesalazine, CFTHs-L, CFTHs-M, and CFTHs-H showed marked improvement in body weight compared to the model group (Figure 4A). In the DSS group, DAI values significantly increased from days 9 to 14, whereas the CFTHs groups demonstrated a slower rise in DAI values during the same period (Figure 4B). Furthermore, colitis induces a pronounced shortening of colon length in mice (Figure 4C). The administration of CFTHs-H notably mitigated colonic shortening.

The spleen, a pivotal immune organ, serves as a key marker of systemic inflammation severity [29]. Consistently, DSS-induced colitis triggered significant splenomegaly when compared to the control group (*p* < 0.001). Notably, this effect was markedly attenuated by CFTHs-H (*p* < 0.05) (Figure 4D).

H&E staining results were evaluated based on epithelial cell damage, crypt swelling, disruption, and inflammatory cell infiltration. The H&E staining displayed that mice in the control group demonstrated preserved colonic architecture, featuring abundant goblet cells, an intact mucosal layer, a thin muscularis, neatly arranged glands, and no inflammatory cell infiltration [30]. In contrast, the colons of the DSS group revealed marked damage to the epithelial surface, thickened edema in the muscularis propria, a reduced number of colonic crypts, the absence of colonic cells in the cups, and enhanced inflammatory cell infiltration. Conversely, the mesalazine and CFTHs treatment groups exhibited a reduced numbers of infiltrating cells, a lesser degree of glandular and crypt damage, repaired partial mucosal injury, and a significant reversal of colonic tissue damage (Figure 4E). In summary, these findings highlight the efficacy of CFTHs in significantly mitigating the symptoms of DSS-induced colitis in mice.

### 3.3. Anti-Inflammatory and Immunomodulatory Effects of CFTHs

**Figure 5:**
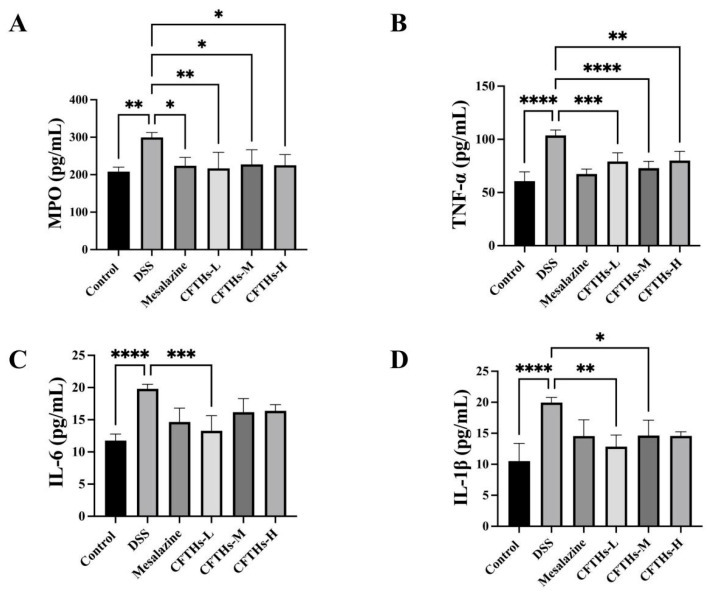
Inflammatory factors and MPO levels in the DSS colitis mouse model. (**A**) MPO; (**B**) TNF-α; (**C**) IL-6; (**D**) IL-1β. Vertical bars represent the standard deviation (n = 10); statistical analyses were conducted using one-way ANOVA followed by Tukey’s post hoc test. “*” denotes statistically significant differences with * *p* < 0.05, ** *p* < 0.01, *** *p* < 0.001, and **** *p* < 0.0001.

MPO is a significant biomarker of inflammation, primarily produced by neutrophils [31]. A key characteristic of chronic colitis is the infiltration of neutrophils. Upon exposure to external stimuli, neutrophils can accumulate in large quantities, subsequently releasing MPO. This enzyme catalyzes the oxidation of chloride ions to produce hypochlorous acid, which destroys various target substances, induces the production of pro-inflammatory factors, and ultimately contributes to inflammation and the elimination of microorganisms within phagocytic cells. Elevated expression of MPO has been observed in the colonic tissues of mice treated with DSS, whereas treatment with CFTHs resulted in a decrease in MPO expression (Figure 5A). This finding indicates that CFTHs effectively inhibit neutrophil chemotaxis and activation, thereby reducing mucosal damage caused by inflammatory cell infiltration [32].

To evaluate the anti-inflammatory effects of CFTHs, key inflammatory cytokines were measured [33]. As shown in Figure 5, DSS induction led to a marked elevation in the colonic levels of these cytokines compared to the controls (*p* < 0.0001). In contrast, CFTHs intervention dramatically decreased the levels of TNF-α, IL-6, and IL-1β. Patients with UC often exhibit overexpression of inflammatory factors in their intestinal tissue; the higher the expression level of these pro-inflammatory factors, the more severe the disease [34]. Mice in the model group developed the most severe colitis, exhibiting the highest levels of pro-inflammatory cytokines. This finding aligns with the results of the present study. The upregulation of pro-inflammatory factors compromises intestinal epithelial integrity, thereby increasing gut permeability. Consequently, this can result in the translocation of certain intestinal bacteria and subsequent intestinal dysfunction. Therefore, CFTHs intervention may inhibit the elevation of pro-inflammatory cytokine levels and protect the intestinal barrier, ultimately maintaining the balance of the gut microbiome.

### 3.4. CFTHs Modulated the Gut Microbiota

**Figure 6:**
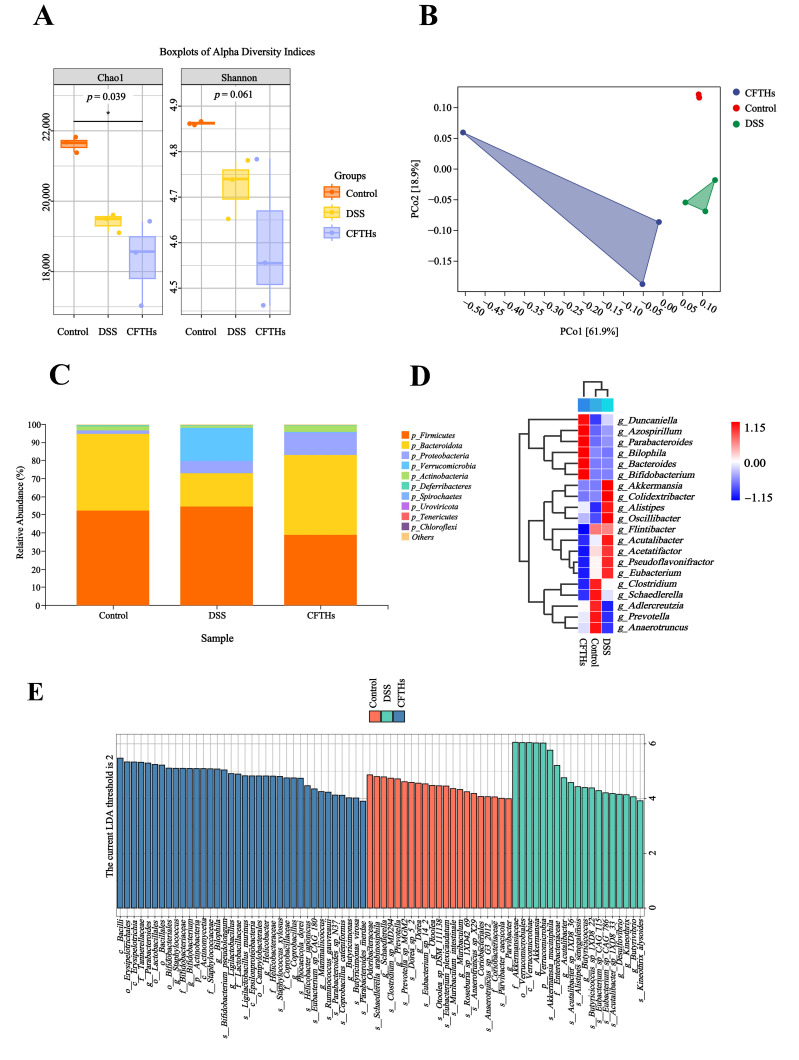
The influence of CFTHs on the gut microbiota in a murine DSS-colitis model. (**A**) Alpha-diversity metrics (Chao 1 and Shannon); (**B**) PCoA; (**C**) microbial makeup at the phylum level; (**D**) heatmap illustrating genus-level species composition. (Color gradient: red for positive correlation, blue for negative correlation; intensity reflects correlation strength); (**E**) linear discriminant analysis Effect Size (LEfSe) analysis of gut microbial composition across the three experimental groups (n = 3). “*” denotes statistically significant differences with * *p* < 0.05.

Gut microbial composition and diversity are key determinants that significantly influence the progression of UC. Some researchers have reported a significant reduction in gut microbial diversity among patients with UC [35]. To investigate the influence of CFTHs on gut microbiota in colitis, fecal metagenomic sequencing was monitored. Alpha diversity, assessed using the Chao1 (richness) and Shannon (diversity/evenness) indices, showed no significant differences across the control, DSS, and CFTHs-treated groups (Figure 6A). The experimental results demonstrated that the administration of CFTHs did not induce significant alterations in either quantitative parameters or biodiversity indices of intestinal microbial communities in colitis models, thereby indicating that the therapeutic effects on colonic inflammation observed during the investigation were likely independent of modulation of the gut microbiome. Consequently, further analysis was conducted to assess the alterations in gut microbiota using beta diversity metrics. Principal Coordinates Analysis (PCoA) based on Bray–Curtis distance revealed a clear separation among the Control, DSS, and CFTHs groups. This indicated that DSS induced a significant alteration in gut microbiota composition, whereas CFTHs facilitated a beneficial structural reorganization of the gut microbiota (Figure 6B).

This study assessed inter-group differences in the murine gut microbiota across two taxonomic levels: phylum and genus. Taxonomic annotation of the fecal microbiota revealed that *Firmicutes* and *Bacteroidetes* comprised approximately 70% of the total bacterial community based on relative abundance and were predominant in the gut microbiota of all groups (Figure 6C). Additionally, the DSS group was enriched in *Firmicutes* and *Verrucomicrobia* compared to the control group, whereas levels of *Bacteroidetes* and *Actinobacteria* dropped. Notably, CFTHs treatment reversed the trends observed in these bacterial populations within the DSS group. To delineate the taxonomic disparities within the microbial communities across the experimental cohorts, a phylogenetic characterization analysis was conducted on the 20 most prevalent genera, selected based on relative abundance thresholds (Figure 6D). Distinct shifts in gut microbiota were observed in DSS compared to the controls. Specifically, the study found a significant increase in genera such as *Akkermansia*, *Oscillibacter*, *Alistipes, Colidextribacter*, *Acetatifactor*, and *Acutalibacter*, while taxa including *Anaerotruncus*, *Adlercreutzia*, *Schaedlerella*, and *Prevotella* exhibited a notable decrease. The evidence suggests that the dysregulated overgrowth of *Acutalibacter* may induce a pro-inflammatory cascade within the intestinal microenvironment. Experimental evidence from murine models demonstrates that the pathogenic activity of this bacterium correlates with spontaneous mucosal inflammation, closely mimicking the clinical features of ulcerative colitis, particularly in terms of histological alterations and cytokine profiles. These mechanistic insights offer a perspective on the possible contribution of *Acutalibacter* in the pathogenesis of chronic intestinal inflammation, suggesting that it may serve as a microbial trigger for the development and progression of colonic inflammatory diseases [36]. Additionally, experimental findings have substantiated a significant depletion of *Oscillibacter* colonization following phloretin therapy. Multivariate regression modeling has demonstrated a pathogenic synergism between the accumulation of *Oscillibacter* biomass and the dysregulated expression of intestinal pro-inflammatory mediators, particularly emphasizing the transcriptional activation of IL-6 and IL-1β [37]. Emerging evidence suggests that *Oscillibacter* exhibits a complex interaction profile within the gut microbiota, with specific correlations observed across various bacterial taxa. Notably, it demonstrates a positive correlation with *Colidextribacter* [38]. This association highlights the potential role of *Oscillibacter* in shaping microbial community structures and modulating metabolic pathways within the gut ecosystem. Phylogenetic clustering analysis revealed substantial convergence between the CFTHs intervention group and untreated controls, with both cohorts maintaining comparable phylogenetic profiles. Collectively, our data supported the potential of CFTHs to mitigate the imbalance of gut microbiota ecology in UC models and warranted further exploration in the future. Comparative microbiota profiling revealed a significant enrichment of taxonomically discriminative genera, including *Staphylococcus*, *Bilophila* and *Bacteroides*, in the CFTHs cohort, establishing a phylogenetic divergence from the control group. *Bacteroides* is closely associated with anti-inflammatory effects. *Bacteroides*, a core beneficial microbiota in the gut, ferment dietary fibers to produce short-chain fatty acids (SCFAs) such as acetic acid and propionic acid. These metabolites play a crucial role in maintaining the integrity of the intestinal barrier, minimizing the entry of pathogens and harmful substances into the bloodstream, suppressing the release of pro-inflammatory cytokines, and promoting the differentiation of regulatory T cells, thereby alleviating inflammation. Research has demonstrated that oral supplementation with *Bacteroides salyersiae* CSP6 exhibits protective effects against DSS-induced colitis in murine models. Specifically, this bacterial strain was found to reverse the pathological mucosal injury caused by DSS. Mechanistic investigations revealed that the administration of *Bacteroides salversiae* CSP6 significantly elevated fecal levels of SCFAs and secondary bile acids–bioactive metabolites with well-established anti-inflammatory properties. This therapeutic outcome was mediated through structural remodeling of the colonic microbiota composition, particularly enhancing the abundance of beneficial commensal species [39].

For the identification of phylogenetic biomarkers across three experimental cohorts, computational biomarker discovery was performed on metagenomic sequencing datasets utilizing the LEfSe algorithm, which facilitated the statistical extraction of biosignatures from taxonomically distinct bacterial taxa, as depicted in Figure 6E. The phylogenetic characterization of microbial consortia under controlled conditions revealed that *g-Prevotella*, *s-Eubacterium*, *s-Anaerotruncus*, and *s-Muribaculum intestinale* served as signature taxa within the control group, demonstrating biomarkers of ecological homeostasis. Conversely, colitic specimens exhibited a dominance of dysbiotic consortia, characterized by *g-Akkermansia*, *g-Acutalibacter*, *s-Alistipes*, and *g-Desulfovibrio*, delineating a microbial architecture enriched with pathobionts. Notably, *Desulfovibrio* positivity was significantly elevated in both acute and chronic UC across multiple regions of the colon [40]. Research indicates that *Desulfovibrio* vulgaris flagellin binds to LRRC19, a cell surface receptor, triggering the release of inflammation-inducing proteins such as IL-6 and TNF-a. This mechanism exacerbates UC by intensifying gut inflammation [41]. *Akkermansia muciniphila* is generally regarded as an anti-inflammatory probiotic that alleviates metabolic inflammation by enhancing gut barrier function. However, under specific conditions, such as Salmonella infection or in IL10 gene-deficient mice, it may excessively degrade the mucus layer, exposing epithelial cells and provoking immune responses, thereby exhibiting pro-inflammatory properties. Studies have shown that *Alistipes* exhibits abnormal abundance levels in patients with inflammatory bowel disease. It may metabolize tryptophan to generate indole derivatives, which could indirectly activate inflammatory pathways [42]. Additionally, the gut microbiota in the CFTHs group was predominantly characterized by *g-Parabacteroides*, *o-Lactobacillales*, *g-Bifidobacterium*, and *g-Butyricimonas*. Evidence indicates that specific strains of *Parabacteroides* can reduce damage to intestinal epithelial cells, thereby mitigating inflammation associated with bacterial migration. This finding suggests that the unique microbial composition of the CFTHs group may play a significant role in the treatment of inflammation. The *Lactobacillales* order, including genera such as *L. acidophilus* and *L. Johnsonii*, can markedly inhibit pro-inflammatory cytokine expression [43]. Furthermore, studies have indicated that treatment with *Lactobacillales* significantly enhances the prevalence of favorable bacteria, including *Muribaculaceae* and *Bifidobacterium*, while concurrently reducing pro-inflammatory lipopolysaccharide levels [44]. *Bifidobacterium*, a well-known probiotic, alleviates intestinal inflammation by modulating gut microbiota, enhancing barrier function, suppressing inflammatory responses, and regulating metabolism. Research has demonstrated that heat-killed *Bifidobacterium bifidum* B1628 (HB1628) mitigates DSS-induced colitis by modulating serum levels of pro-inflammatory factors (IL-1β, TNF-α) and anti-inflammatory factors (IL-13), while significantly enriching metabolic pathways such as tryptophan biosynthesis [45]. The genus *Butyricimonas* primarily produces butyrate, which directly suppresses inflammatory responses and protects the intestinal barrier. Butyrate is crucial for maintaining intestinal barrier integrity and inhibiting the release of inflammatory factors [46].

*Butyricimonas* and *Oscillibacter* are not adept at directly utilizing dietary fiber; instead, they rely on upstream bacterial communities such as *Bifidobacterium* and *Lactobacillales*. These communities produce lactic acid and acetic acid as substrates through the acetyl CoA pathway or the butyrate kinase pathway, ultimately leading to the production of butyric acid. This synergistic alliance of beneficial bacteria suppresses the growth of potential pathogens and harmful bacteria, such as *Desulfovibrio*, by competing for nutrients and occupying intestinal space. Consequently, this competition controls their population at lower levels and prevents the excessive production of hydrogen sulfide, which can damage the intestinal barrier. Butyric acid directly inhibits key pro-inflammatory signaling pathways, such as NF-κB, thereby downregulating the production of cytokines including TNF-α, IL-6, and IL-1β. Furthermore, butyric acid serves as the primary energy source for colonic epithelial cells, strengthening the intestinal barrier and preventing endotoxins from entering the bloodstream, which could lead to systemic inflammation. Propionic acid, primarily produced by Bacteroides, can exert anti-inflammatory effects in peripheral tissues, such as the liver and immune cells, once it enters the bloodstream. In addition, SCFAs promote the function of regulatory T cells, which maintain immune tolerance. In summary, our metagenomic sequencing successfully characterized the impact of CFTHs on gut microbiota composition, revealing a shift towards a healthier microbiota structure.

### 3.5. CFTHs Altered the Functional Profile of the Gut Microbial Metagenome

**Figure 7:**
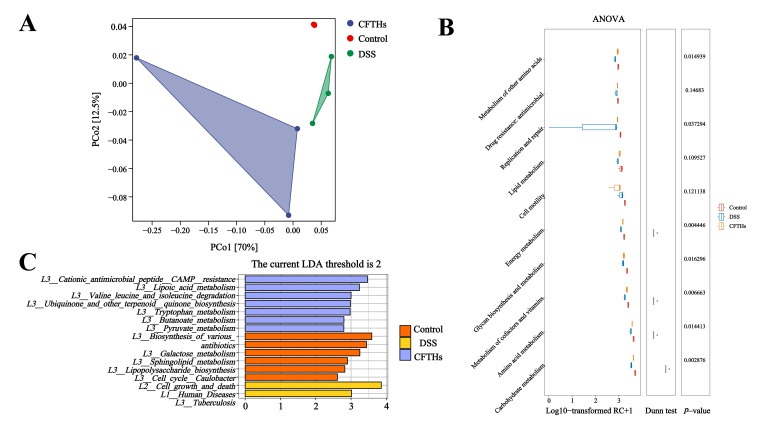
Impact of CFTHs on the gut functional metagenome in a murine DSS-colitis model. (**A**) KO-based beta-diversity; (**B**) Comparative profiling of level-2 KEGG pathways; (**C**) LEfSe biomarker discovery for level-3 KEGG pathways (n = 3).

Given the intrinsic correlation between structural shifts in gut microbiota composition and its metabolic potential, the functional implications of CFTHs supplementation were systematically evaluated. Beta-diversity analysis, utilizing Bray–Curtis PCoA, revealed distinct clustering patterns among experimental groups when examining Kyoto Encyclopedia of Genes and Genomes (KEGG) Orthology (KO) profiles. This phylogenetic segregation at the molecular function level suggested fundamental metabolic reprogramming induced by CFTHs intervention (Figure 7A). A comparative analysis of KEGG level two annotations revealed substantial alterations in metabolic pathways within DSS-induced colitis models (Figure 7B). Notably, the ten metabolic pathways displaying the highest gene enrichment scores demonstrated a significant attenuation of microbial functional capacity under DSS treatment. Mechanistically, the administration of CFTHs restored microbial functional across all perturbed pathways, effectively reversing DSS-mediated metabolic suppression through global functional recalibration. However, differential significance was not universally observed across all pathways. To delineate functionally distinct pathways, LEfSe analysis was conducted across three hierarchical levels of KEGG classification (Figure 7C), identifying six metabolic pathways that met the LDA threshold of >2. Subsequent characterization revealed that the control group was predominantly involved in: (1) biosynthesis of various antibiotics, (2) galactose metabolism, (3) sphingolipid metabolism, (4) lipopolysaccharide biosynthesis, (5) cell cycle in *Caulobacter*, and (6) cell growth and death. Comparative analysis revealed distinct pathway signatures between experimental groups, with the model group demonstrating a predominant association with disease-related metabolism, particularly tuberculosis pathogenesis pathways (LDA > 2). Conversely, the CFTHs group exhibited a characteristic enrichment across seven functional clusters: (1) cationic antimicrobial peptide CAMP resistance, (2) lipoic acid metabolism, (3) valine leucine and isoleucine degradation, (4) ubiquinone and other terpenoid quinone biosynthesis, (5) tryptophan metabolism, (6) butanoate metabolism, and (7) pyruvate metabolism. Butyrate, a type of SCFA in the gut, is primarily synthesized through microbial fermentation of dietary fiber by intestinal microbiota. It plays critical roles in nourishing colonic epithelial cells, maintaining intestinal barrier integrity, and suppressing inflammatory responses. In patients with IBD, gut microbial dysbiosis may lead to diminished butyrate production, which subsequently disrupts barrier function, elevates intestinal permeability, and exacerbates inflammation. Clinical trials have indicated that exogenous butyrate administration significantly attenuates intestinal inflammation in experimental colitis models while concurrently promoting mucosal repair mechanisms in UC patients, as evidenced by endoscopic and histological improvements [47]. Tryptophan, an essential amino acid, undergoes metabolic processing through both the host-mediated kynurenine pathway and the microbiota-dependent indole pathway. Key tryptophan-derived metabolites, such as indolepropionic acid, function as ligands for the aryl hydrocarbon receptor (Ah), modulating immune responses and maintaining intestinal homeostasis. In IBD, dysregulated tryptophan metabolism is associated with impaired Ah signaling [48]. Emerging evidence suggests that UC patients exhibit reduced circulating levels of tryptophan metabolites, which correlate positively with clinical disease activity indices.

### 3.6. Modulation of the Metabolome in Colitis by CFTHs

**Figure 8:**
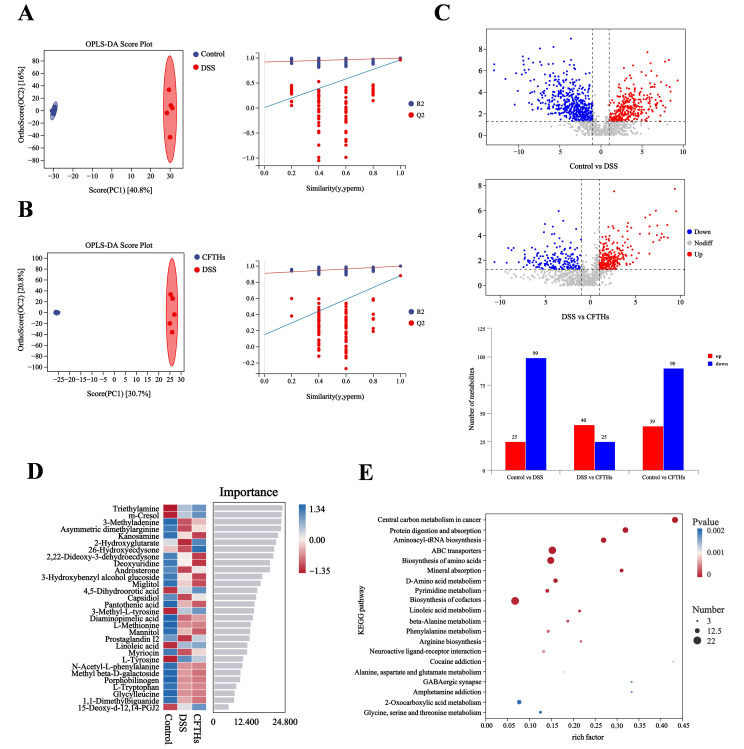
Impact of CFTHs on microbial metabolites in colitis (**A**,**B**) OPLS-DA with permutation testing; (**C**) Volcano plots of differential metabolites with bar graphs depicting the influence of CFTHs. In the volcano plot, the horizontal axis represents the log 2 value of the fold change (FC), while the vertical axis represents the −log 10 value of the significance *p*-value. Metabolites that meet the criteria of FC > 1 and *p*-value < 0.05 are highlighted in red, whereas those with FC < 1 and *p*-value < 0.05 are indicated in blue. Non-significantly differentiated metabolites are displayed in gray; (**D**) Top 30 metabolites identified by machine learning in triple-group comparisons; (**E**) Comparative pathway enrichment analysis (n = 5).

To investigate the metabolic perturbations induced by DSS and the therapeutic effects of CFTHs, a comparative metabolomic analysis was conducted among three experimental groups: Control, DSS-treated, and CFTHs-intervened mice (Figure 8A,B). The analysis of colon tissue metabolomics revealed distinct clustering patterns in the colonic tissue metabolomes, with a clear separation between the DSS and both the control and CFTHs groups. This differential clustering demonstrates significant metabolic reprogramming induced by the development of colitis, followed by normalization through CFTHs intervention. Additionally, to elucidate the CFTHs-mediated regulation of gut microbiota-derived metabolism, the colonic metabolomic profiles were analyzed using orthogonal partial least squares-discriminant analysis (OPLS-DA). Statistical validation confirmed the model’s accuracy, with all predictive performance scores (Q2) from the test being significantly lower than those from the original dataset, indicating no overfitting. This validation protocol confirmed the discriminative power of the OPLS-DA model in detecting CFTHs-induced metabolic remodeling within the colitis microenvironment. Quantitative profiling revealed a DSS-induced metabolic imbalance characterized by a dose-dependent dysregulation of 124 metabolites (Figure 8C). In our study, a pronounced elevation was observed in 25 metabolites, particularly triethylamine, derived from gut microbiota, and methylmalonic acid (MMA), which was associated with energy metabolism. Conversely, 99 metabolites exhibited significant suppression, including diaminopimelic acid and 7-methylguanine, which are microbial–mammalian co-metabolites. These findings suggest an impairment in microbial–host co-metabolism during the pathogenesis of colitis. MMA, a clinical biomarker for functional vitamin B12 deficiency, plays a critical role in propionate metabolism, necessitating the activity of adenosylcobalamin-dependent methylmalonyl-CoA mutase. Impaired vitamin B12 metabolism results in diminished MMA conversion capacity, leading to its abnormal accumulation. Our findings align with clinical epidemiological studies that demonstrate elevated MMA levels in patients with IBD, reflecting impaired utilization of vitamin B12 [49]. The prevalence of deficiency exhibits distinct pathoanatomical patterns: patients with Crohn’s disease display deficiency rates between 15% and 33%, escalating to 40% to 70% with ileal involvement, while those with UC show a prevalence of 5% to 16%, predominantly in patients with pancolitis. The diagnostic approach utilizing both holotranscobalamin quantification and MMA analysis demonstrated that intestinal inflammation was an autonomous determinant of cobalamin homeostasis. This research delineated the dual role of MMA in the pathogenesis of colitis [50]. Metabolomic profiling in DSS-induced colitis mice demonstrated that CFTHs treatment induced significant upregulation of 40 bioactive molecules, notably including prostaglandin I2, gamma-linolenic acid, and palmitoylethanolamide, concomitant with the suppression of 25 metabolites, including hydroquinone, L-arginine, and succinic acid.

Our analysis identified a set of top-ranked differential metabolites, including EPA (d5), gamma-linolenic acid, ethyl icosapentate, 2-hydroxyglutarate, and prostaglandin I2 (Figure 8D). As an ω-3 polyunsaturated fatty acid, EPA significantly reduced inflammatory responses by inhibiting the production of pro-inflammatory mediators such as prostaglandin E2 (PGE2) and leukotriene B4 (LTB4), while activating anti-inflammatory receptors (GPR120) and suppressing the NF-κB signaling pathway. It is commonly used in the management of cardiovascular diseases and chronic inflammatory conditions [51]. Ethyl icosapentate, an ethyl ester derivative of EPA, functions as a lipid-regulating drug. By elevating blood EPA concentrations, it exerted anti-inflammatory effects identical to those of EPA, particularly in mitigating atherosclerosis-associated inflammation [52]. Gamma-linolenic acid, a member of the ω-6 fatty acid family, is metabolized to dihomo-gamma-linolenic acid. In the DSS-induced colitis mouse model, the enrichment of DGLA-related metabolic pathways suggests that it may alleviate colon injury by regulating lipid oxidation and inflammatory signaling pathways, such as the NLRP3 inflammasome [53]. The anti-inflammatory effect of 2-hydroxyglutarate is partially mediated by the suppression of inflammatory protein expression triggered by lipopolysaccharide (LPS). This suppression suggests that 2-hydroxyglutarate may alleviate gut inflammation by reducing essential inflammatory triggers [54]. Moreover, key metabolic pathways were identified across all groups, as illustrated in Figure 8E. The significantly altered pathways encompassed several functional domains, including central carbon metabolism in cancer, protein digestion and absorption, and amino acid/nucleotide metabolism.

The three metabolic pathways include central carbon metabolism in cancer, the biosynthesis of amino acids, along with mineral absorption and protein digestion and absorption. These pathways are fundamental to cellular life activities, and their upregulation indicates enhanced metabolic activity in immune cells. L-arginine, a core product of protein digestion and amino acid biosynthesis, provides a material basis for effective immune responses. The abundance of L-arginine directly supports the synthesis of NO, thereby mediating vasodilation and inhibiting inflammation. GLA promotes the production of anti-inflammatory PGI2 through a metabolic shift. Therefore, a composite formula with L-arginine and GLA as the core components can be specifically designed for the management of chronic inflammation. Additionally, 2-Hydroxyglutaric acid, recognized as a potential epigenetic regulator, may affect gene expression in immune cells by influencing DNA and histone methylation, thereby leading to a tendency towards an anti-inflammatory phenotype. Identifying natural ingredients that can safely regulate epigenetics is the research and development direction for CFTHs to evolve into the next generation of high-end health products. Therefore, the untargeted metabolomics approach has effectively profiled the global shifts in the host metabolome induced by CFTHs, identifying key metabolic pathways that are potentially central to their therapeutic mechanisms.

### 3.7. Integrating Multi-Omics: Correlations Among Gut Flora, Metabolites, and Host Physiology

**Figure 9:**
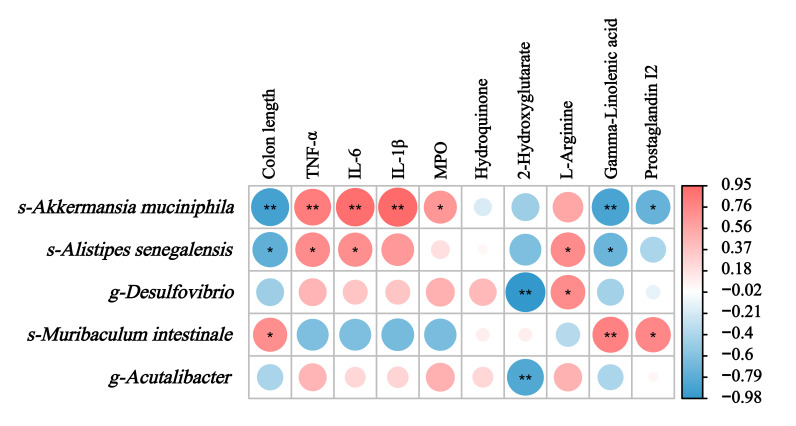
Interrelationships among gut flora, metabolites, and clinical parameters. “*” denotes a statistical correlation, with * *p* < 0.05 and ** *p* < 0.01.

Spearman’s correlation analysis was conducted to evaluate potential associations among colitis-related clinical parameters, microbial community profiles, and metabolic alterations. As illustrated in Figure 9, the study identified a positive association between the probiotic strain *Muribaculum* and two physiological indicators: intestinal colon length and the bioactive compounds gamma-linolenic acid and prostaglandin I2. Notably, this microbial species demonstrated an inverse relationship with inflammatory markers. Conversely, the pathobionts *Akkermansia muciniphila* and *Alistipes senegalensis* displayed inverse associations with the aforementioned beneficial strain, specifically exhibiting contrasting patterns in colon length measurements and inflammatory biomarker profiles. Our metabolomic correlation analysis further revealed distinct interaction patterns: the amino acid derivative L-arginine was positively associated with *Alistipes senegalensis* and sulfate-reducing *Desulfovibrio*, whereas exhibiting an inverse relationship with the beneficial *Muribaculum*. Particularly striking were the universal inhibitory effects of 2-hydroxyglutarate against all pathobionts characterized in this investigation. These results suggest that harmful gut bacteria compete with one another, and as demonstrated in our findings, CFTHs may mitigate colitis via remodeling the gut microbial and metabolic environment. The relationship between microbial taxa, metabolites, and host cytokines is established through correlation analysis; however, future fecal transplantation experiments are necessary to validate these potential mechanistic pathways.

## 4. Conclusions

Our investigation defines CFTHs as structurally robust polypeptides that demonstrate optimal biopharmaceutical characteristics, exhibiting minimal surface hydrophobicity and superior aqueous solubility. Notably, enzymatic hydrolysate analysis revealed that favourzyme-processed CFTHs possess an ultra-narrow molecular weight profile, with 81.25% of peptide fractions retaining masses below 1000 Da, which is a critical quality attribute for bioactive peptide therapeutics. In murine models of UC, the administration of CFTHs significantly attenuated DSS-induced pathological manifestations, as evidenced by both macroscopic improvements (reduced DAI scores) and histological confirmation of decreased intestinal mucosal damage. The therapeutic efficacy was further corroborated by systemic anti-inflammatory effects, including normalization of the spleen index and downregulation of key pro-inflammatory cytokines. Compared to previous studies on the by-products of *Cucumaria frondosa*, our research offers a novel integration of metagenomics and metabolomics to investigate their correlation and potential mechanisms of action in colitis. This investigation employed integrated metagenomic and metabolomic profiling to demonstrate that CFTHs may alleviate intestinal inflammation in a murine colitis model by comprehensively regulating the microbial community. The intervention effect appears to be related to the reconstruction of microbial communities, primarily characterized by the enrichment of probiotics such as *Muribaculum*, and a significant reduction in potential pathogenic bacteria such as *Akkermansia muciniphila*, *Alistipes senegalensis*, *Desulfovibrio*, and *Acutalibacter*. Additionally, CFTHs may activate microbiota-derived metabolic networks that are crucial for maintaining intestinal homeostasis. Metabolomic features further suggest that CFTHs exhibit superior metabolic remodeling capabilities compared to controls, with their role in altering gut microbiota potentially contributing to the decreased relative abundance of L-arginine. Furthermore, the treatment increased levels of anti-inflammatory metabolites, including gamma-linolenic acid and prostaglandin I2, which were speculated to mitigate gut inflammation by regulating amino acid metabolism. Collectively, the demonstrated stability and bioactivity of CFTHs position them as promising candidates for contemporary colitis treatment, particularly in the context of various functional foods aimed at promoting gut health. The potent bioactivity, combined with the sustainable sourcing of tentacles as a marine by-product, underscores their commercial viability. However, it is important to acknowledge a limitation of this study: the inherent physiological and immunological differences between mice and humans, as well as potential variations in disease pathogenesis. This study utilized a murine model, and further research, including clinical trials, is warranted to validate the efficacy and safety of CFTHs in humans.

## Figures and Tables

**Table 1:** Optimal hydrolysis conditions for hydrolysates prepared using various proteases.

Proteases	Temperature (°C)	pH	Solid–Liquid Ratio (*w*/*v*)	Proteases Addition (U/g Protein)	Time (h)	Degree of Hydrolysis (%)
Trypsase	55	8.0	1:4	9500	4	14.88 ± 0.54
Flavourzyme	40	5.8	1:6	10,000	7	40.47 ± 0.86
Alcalase	55	9.5	1:8	8500	7	25.87 ± 0.91
Neutrase	45	7.2	1:10	7500	5	13.90 ± 0.86

**Table 2:** DAI scoring system utilized for assessing DSS-induced colitis in mice.

Score	Diarrhea	Stool Bleeding	Body Weight Loss (%)
0	normal	normal	0–1
1	slightly loose stools	little reddish	1–5
2	loose stools	brown reddish color	5–10
3	diarrhea	visible blood	10–20
4	watery diarrhea	rectal bleeding	>20

## Data Availability

The data presented in this study are available on request from the corresponding authors.

## Abbreviations

The following abbreviations are used in this manuscript:

CFTHs	*Cucumaria frondosa* tentacles hydrolysates
DSS	Dextran sulfate sodium
IBD	Inflammatory bowel disease
UC	Ucerative colitis
CD	Crohn’s disease
ELISA	Enzyme-linked immunosorbent assay
TNF-α	Tumor necrosis factor-alpha
IL-6	Interleukin-6
IL-1β	Interleukin-1 beta
MPO	Myeloperoxidase
ANS	1-anilino-8-naphthalenesulfonate
PBS	Phosphate-buffered saline
HPLC	High-performance liquid chromatography
CFTHs-L	*Cucumaria frondosa* tentacles hydrolysates low-dose group
CFTHs-M	*Cucumaria frondosa* tentacles hydrolysates medium-dose group
CFTHs-H	*Cucumaria frondosa* tentacles hydrolysates high-dose group
DAI	Disease activity index
H&E	Hematoxylin-eosin
SCFAs	Short-chain fatty acids
PCoA	Principal Coordinates Analysis
KEGG	Kyoto Encyclopedia of Genes and Genomes
KO	Orthology
OPLS-DA	Orthogonal partial least squares-discriminant analysis
MMA	Methylmalonic acid
QC	Quality control
UPLC	Ultra-performance liquid chromatograph
SD	Standard deviation
Ah	Aryl hydrocarbon receptor
LPS	Lipopolysaccharide
FC	Fold change

## References

[1] Hossain A., Dave D., Shahidi F. (2020). Northern Sea Cucumber (*Cucumaria frondosa*): A Potential Candidate for Functional Food, Nutraceutical, and Pharmaceutical Sector. Mar. Drugs.

[2] Vu D.T., Falch E., Elvevoll E.O., Jensen I.J. (2023). Enzymatic Hydrolysis of Orange-Footed Sea Cucumber (*Cucumaria frondosa*)-Effect of Different Enzymes on Protein Yield and Bioactivity. Foods.

[3] Fagbohun O.F., Rollins A., Mattern L., Cipollini K., Rupasinghe H.V. (2024). Frondoside A of *Cucumaria frondosa* (Gennerus, 1767): Chemistry, biosynthesis, medicinal applications, and mechanism of actions. J. Pharm. Pharmacol..

[4] Li M., Chen J., Wang Q., Liu C., Song W., Sun L. (2025). Characteristics, Antioxidant Activity Stability, and Anti-Fatigue Activity of Hydrolysates from *Cucumaria frondosa* Tentacles. Molecules.

[5] Xu P.X., Luo S.W., Song J.F., Dai Z.Q., Li D.J., Wu C.E. (2023). Effect of sodium alginate-based hydrogel loaded with lutein on gut microbiota and inflammatory response in DSS-induced colitis mice. Food Sci. Hum. Well..

[6] Albtoush N., Queisser K.A., Zawerton A., Lauer M.E., Beswick E.J., Petrey A.C. (2023). TSG6 hyaluronan matrix remodeling dampens the inflammatory response during colitis. Matrix Biol..

[7] Feng Y.Q., Chen S.M., Song Y.T., Liu S.H., Duan Y.Q., Cai M.H., Kong T.Y., Zhang H.H. (2024). A novel *Sagittaria sagittifolia* L. polysaccharides mitigate DSS-induced colitis via modulation of gut microbiota and MAPK/NF-κB signaling pathways. Int. J. Biol. Macromol..

[8] Liu Y.H., Liu G., Fang J. (2024). Progress on the mechanisms of *Lactobacillus plantarum* to improve intestinal barrier function in ulcerative colitis. J. Nutr. Biochem..

[9] Zhang X.J., Li A., Wang Y.Y.F., Wang J., Zhang B.W., Zhang Y., Liu J.M., Wang S. (2024). D-Psicose intake exacerbates dextran sulfate sodium-induced colitis in mice through alteration in the gut microbiota and dysfunction of mucosal barrier. Food Sci. Hum. Well..

[10] Senadheera T.R.L., Hossain A., Dave D., Shahidi F. (2022). In Silico Analysis of Bioactive Peptides Produced from Underutilized Sea Cucumber By-Products-A Bioinformatics Approach. Mar. Drugs.

[11] Sivaraman K., Shanthi C. (2022). Purified fish skin collagen hydrolysate attenuates TNF-α induced barrier dysfunction in-vitro and DSS induced colitis in-vivo model. Int. J. Biol. Macromol..

[12] Ambigaipalan P., Shahidi F. (2017). Bioactive peptides from shrimp shell processing discards: Antioxidant and biological activities. J. Funct. Foods.

[13] Li Y.J., Zhang Y.Y., Tuo Y., You H.X., Li J.L., Wang L.Y., Liu X.B., Ding L. (2023). Quinoa protein and its hydrolysate ameliorated DSS-induced colitis in mice by modulating intestinal microbiota and inhibiting inflammatory response. Int. J. Biol. Macromol..

[14] Mao J., Zhao Y.J., Wang L.C., Wu T., Jin Y., Meng J., Zhang M. (2023). Sea Cucumber Peptide Alleviates Ulcerative Colitis Induced by Dextran Sulfate Sodium by Alleviating Gut Microbiota Imbalance and Regulating miR-155/SOCS1 Axis in Mice. Foods.

[15] Subramanya S.B., Chandran S., Almarzooqi S., Raj V., Al Zahmi A.S., Al Katheeri R.A., Al Zadjali S.A., Collin P.D., Adrian T.E. (2018). Frondanol, a Nutraceutical Extract from *Cucumaria frondosa*, Attenuates Colonic Inflammation in a DSS-Induced Colitis Model in Mice. Mar. Drugs.

[16] Zhang Q.T., Tu Z.C., Xiao H., Wang H., Huang X.Q., Liu G.X., Liu C.M., Shi Y., Fan L.L., Lin D.R. (2014). Influence of ultrasonic treatment on the structure and emulsifying properties of peanut protein isolate. Food Bioprod. Process..

[17] Yu X., Chen Y.A., Qi Z.G., Chen Q., Cao Y.J., Kong Q.S. (2023). Preparation and identification of a novel peptide with high antioxidant activity from corn gluten meal. Food Chem..

[18] Xiang X.W., Zhou X.L., Wang R., Shu C.H., Zhou Y.F., Ying X.G., Zheng B. (2021). Protective Effect of Tuna Bioactive Peptide on Dextran Sulfate Sodium-Induced Colitis in Mice. Mar. Drugs.

[19] Ullah H., Deng T., Ali M., Farooqui N.A., Alsholi D.M., Siddiqui N.Z., Rehman A.U., Ali S., Ilyas M., Wang L. (2023). Sea Conch Peptides Hydrolysate Alleviates DSS-Induced Colitis in Mice through Immune Modulation and Gut Microbiota Restoration. Molecules.

[20] Gao J.H., Li L.X., Zhao D., Wang X., Xia Y.A., Li B., Liu C., Zuo X.L. (2022). Tilapia skin peptides, a by-product of fish processing, ameliorate DSS-induced colitis by regulating inflammation and inhibiting apoptosis. Front. Nutr..

[21] Feng J.H., Zhang L.N., Tang X., Hu W., Zhou P. (2022). Major yolk protein from sea cucumber (*Stichopus japonicus*) attenuates acute colitis via regulation of microbial dysbiosis and inflammatory responses. Food Res. Int..

[22] Yu F.Z., Wang X.X., Ren H.L., Chang J., Guo J., He Z.Q., Shi R.R., Hu X.Y., Jin Y.Y., Lu S.Y. (2024). *Lactobacillus paracasei* Jlus66 relieves DSS-induced ulcerative colitis in a murine model by maintaining intestinal barrier integrity, inhibiting inflammation, and improving intestinal microbiota structure. Eur. J. Nutr..

[23] Fadimu G.J., Gill H., Farahnaky A., Truong T. (2022). Improving the enzymolysis efficiency of lupin protein by ultrasound pretreatment: Effect on antihypertensive, antidiabetic and antioxidant activities of the hydrolysates. Food Chem..

[24] Pan X., Fang Y., Wang L.L., Xie M.H., Hu B., Zhu Y.Q., Zhao E.M., Pei F., Shen F., Li P. (2019). Effect of enzyme types on the stability of oil-in-water emulsions formed with rice protein hydrolysates. J. Sci. Food Agr..

[25] Wei M.M., Ning C., Ren Y.F., Hu F.Q., Wang M.X., Li W.X. (2024). Characterisation and comparison of enzymatically prepared donkey milk whey protein hydrolysates. Food Chem.-X..

[26] Abdelhedi O., Mora L., Jridi M., Toldrá F., Nasri M. (2024). Proteolysis Coupled with Membrane Separation for the Isolation of Bioactive Peptides from Defatted Smooth Hound Byproduct Proteins. Waste Biomass Valori..

[27] Heffernan S., Giblin L., O’Brien N. (2021). Assessment of the biological activity of fish muscle protein hydrolysates using in vitro model systems. Food Chem..

[28] Wu A.X., Gao Y., Kan R.T., Ren P.F., Xue C.H., Kong B., Tang Q.J. (2023). Alginate Oligosaccharides Prevent Dextran-Sulfate-Sodium-Induced Ulcerative Colitis via Enhancing Intestinal Barrier Function and Modulating Gut Microbiota. Foods.

[29] Lima G.R.D., Machado F.D.F., Perico L.L., de Faria F.M., Luiz-Ferreira A., Brito A., Pellizzon C.H., Hiruma-Lima C.A., Tavares J.F., Barbosa J.M. (2017). Anti-inflammatory intestinal activity of *Combretum duarteanum* Cambess. in trinitrobenzene sulfonic acid colitis model. World J. Gastroenterol..

[30] Dong W.R., Li Y.Y., Liu T.T., Zhou G., Chen Y.X. (2023). Ethyl acetate extract of *Terminalia chebula* alleviates DSS-induced ulcerative colitis in C57BL/6 mice. Front. Pharmacol..

[31] Chen T.Y., Liu J.C., Hang R.Y., Chen Q., Wang D. (2025). Neutrophils: From Inflammatory Bowel Disease to Colitis-Associated Colorectal Cancer. J. Inflamm. Res..

[32] Li M., Lan L.L., Zhang S., Xu Y.J., He W.X., Xiang D., Liu D., Ren X.H., Zhang C.L. (2021). IL-6 downregulates hepatic carboxylesterases via NF-κB activation in dextran sulfate sodium-induced colitis. Int. Immunopharmacol..

[33] Gong S.M., Li M.B., Gao J.R., Huang S.J., Song W.K., Sun L.L. (2025). *Cucumaria frondosa* intestines and ovum hydrolysates intervention ameliorates the symptoms of dextran sulfate sodium-induced colitis by modulating gut microbiota and its metabolites. J. Food Sci..

[34] Nakase H., Sato N., Mizuno N., Ikawa Y. (2022). The influence of cytokines on the complex pathology of ulcerative colitis. Autoimmun. Rev..

[35] Alipour M., Zaidi D., Valcheva R., Jovel J., Martínez I., Sergi C., Walter J., Mason A.L., Wong G.K.S., Dieleman L.A. (2016). Mucosal Barrier Depletion and Loss of Bacterial Diversity are Primary Abnormalities in Paediatric Ulcerative Colitis. J. Crohns Colitis.

[36] Bolsega S., Basic M., Smoczek A., Buettner M., Eberl C., Ahrens D., Odum K.A., Stecher B., Bleich A. (2019). Composition of the Intestinal Microbiota Determines the Outcome of Virus-Triggered Colitis in Mice. Front. Immunol..

[37] Wu M.N., Li P.Z., An Y.Y., Ren J., Yan D., Cui J.Z., Li D., Li M., Wang M.Y., Zhong G.S. (2019). Phloretin ameliorates dextran sulfate sodium-induced ulcerative colitis in mice by regulating the gut microbiota. Pharmacol. Res..

[38] Gu H., Tian Y.W., Xia J.J., Deng X.Y., Chen J., Jian T.Y., Ma J. (2024). Li-Hong Tang alleviates dextran sodium sulfate-induced colitis by regulating NRF2/HO-1 signaling pathway and gut microbiota. Front. Pharmacol..

[39] Dai W., Lv Y.J., Quan M., Ma M.F., Shang Q.S., Yu G.L. (2024). *Bacteroides salyersiae* Is a Candidate Probiotic Species with Potential Anti-Colitis Properties in the Human Colon: First Evidence from an In Vivo Mouse Model. Nutrients.

[40] Rowan F., Docherty N.G., Murphy M., Murphy B., Coffey J.C., O’Connell P.R. (2010). Desulfovibrio Bacterial Species Are Increased in Ulcerative Colitis. Dis. Colon Rectum.

[41] Huang G.X., Zheng Y.L., Zhang N., Huang G.H., Zhang W.J., Li Q.N., Ren X.C. (2024). *Desulfovibrio vulgaris* caused gut inflammation and aggravated DSS-induced colitis in C57BL/6 mice model. Gut Pathog..

[42] Dziarski R., Park S.Y., Kashyap D.R., Dowd S.E., Gupta D. (2016). Pglyrp-Regulated Gut Microflora *Prevotella falsenii*, *Parabacteroides distasonis* and *Bacteroides eggerthii* Enhance and *Alistipes finegoldii* Attenuates Colitis in Mice. PLoS ONE.

[43] Chen L.L., Zou Y.Y., Peng J., Lu F.G., Yin Y.N., Li F.J., Yang J.W. (2015). *Lactobacillus acidophilus* Suppresses Colitis-Associated Activation of the IL-23/Th17 Axis. J. Immunol. Res..

[44] Yao M.F., Lu Y.M., Zhang T., Xie J.J., Han S.Y., Zhang S.B., Fei Y.Q., Ling Z.X., Wu J.J., Hu Y. (2021). Improved functionality of *Ligilactobacillus salivarius* Li01 in alleviating colonic inflammation by layer-by-layer microencapsulation. Npj Biofilms Microbiomes.

[45] Feng C.J., Zhang W.Q., Zhang T., He Q.W., Kwok L.Y., Tan Y., Zhang H.P. (2022). Heat-Killed *Bifidobacterium bifidum* B1628 May Alleviate Dextran Sulfate Sodium-Induced Colitis in Mice, and the Anti-Inflammatory Effect Is Associated with Gut Microbiota Modulation. Nutrients.

[46] Zhang S.S., Nie Q.X., Sun Y.G., Zuo S., Chen C.H., Li S., Yang J.R., Hu J.L., Zhou X.T., Yu Y.K. (2024). *Bacteroides uniformis* degrades β-glucan to promote *Lactobacillus johnsonii* improving indole-3-lactic acid levels in alleviating colitis. Microbiome.

[47] Venegas D.P., De la Fuente M.K., Landskron G., González M.J., Quera R., Dijkstra G., Harmsen H.J.M., Faber K.N., Hermoso M.A. (2019). Short Chain Fatty Acids (SCFAs)-Mediated Gut Epithelial and Immune Regulation and Its Relevance for Inflammatory Bowel Diseases. Front. Immunol..

[48] Pandey H., Jain D., Tang D.W.T., Wong S.H., Lal D. (2024). Gut microbiota in pathophysiology, diagnosis, and therapeutics of inflammatory bowel disease. Intest. Res..

[49] Akbulut S. (2022). An assessment of serum vitamin B12 and folate in patients with Crohn’s disease. Medicine.

[50] Ward M.G., Kariyawasam V.C., Mogan S.B., Patel K.V., Pantelidou M., Sobczynska-Malefora A., Porté F., Griffin N., Anderson S.H.C., Sanderson J.D. (2015). Prevalence and Risk Factors for Functional Vitamin B_12_ Deficiency in Patients with Crohn’s Disease. Inflamm. Bowel Dis..

[51] El Mahdy R.N., Nader M.A., Helal M.G., Abu-Risha S.E., Abdelmageed M.E. (2023). Eicosapentaenoic acid mitigates ulcerative colitis-induced by acetic acid through modulation of NF-κB and TGF-β/EGFR signaling pathways. Life Sci..

[52] Crupi R., Cuzzocrea S. (2022). Role of EPA in Inflammation: Mechanisms, Effects, and Clinical Relevance. Biomolecules.

[53] Yu T.Y., Feng Y.M., Kong W.S., Li S.N., Sun X.J., Zhou G., Xie R.F., Zhou X. (2023). Gallic acid ameliorates dextran sulfate sodium-induced ulcerative colitis in mice via inhibiting NLRP3 inflammasome. Front. Pharmacol..

[54] Han C.J., Zheng J.Y., Sun L., Yang H.C., Cao Z.Q., Zhang X.H., Zheng L.T., Zhen X.C. (2019). The oncometabolite 2-hydroxyglutarate inhibits microglial activation via the AMPK/mTOR/NF-κB pathway. Acta Pharmacol. Sin..

